# Burn size determines the inflammatory and hypermetabolic response

**DOI:** 10.1186/cc6102

**Published:** 2007-08-23

**Authors:** Marc G Jeschke, Ronald P Mlcak, Celeste C Finnerty, William B Norbury, Gerd G Gauglitz, Gabriela A Kulp, David N Herndon

**Affiliations:** 1 Shriners Hospitals for Children, 815 Market Street, Galveston, TX 77550, USA; 2 Department of Surgery, University Texas Medical Branch, Galveston, TX, 77550 USA

## Abstract

**Background:**

Increased burn size leads to increased mortality of burned patients. Whether mortality is due to inflammation, hypermetabolism or other pathophysiologic contributing factors is not entirely determined. The purpose of the present study was to determine in a large prospective clinical trial whether different burn sizes are associated with differences in inflammation, body composition, protein synthesis, or organ function.

**Methods:**

Pediatric burned patients were divided into four burn size groups: <40% total body surface area (TBSA) burn, 40–59% TBSA burn, 60–79% TBSA burn, and >80% TBSA burn. Demographic and clinical data, hypermetabolism, the inflammatory response, body composition, the muscle protein net balance, serum and urine hormones and proteins, and cardiac function and changes in liver size were determined.

**Results:**

One hundred and eighty-nine pediatric patients of similar age and gender distribution were included in the study (<40% TBSA burn, *n *= 43; 40–59% TBSA burn, *n *= 79; 60–79% TBSA burn, *n *= 46; >80% TBSA burn, *n *= 21). Patients with larger burns had more operations, a greater incidence of infections and sepsis, and higher mortality rates compared with the other groups (*P *< 0.05). The percentage predicted resting energy expenditure was highest in the >80% TBSA group, followed by the 60–79% TBSA burn group (*P *< 0.05). Children with >80% burns lost the most body weight, lean body mass, muscle protein and bone mineral content (*P *< 0.05). The urine cortisol concentration was highest in the 80–99% and 60–79% TBSA burn groups, associated with significant myocardial depression and increased change in liver size (*P *< 0.05). The cytokine profile showed distinct differences in expression of IL-8, TNF, IL-6, IL-12p70, monocyte chemoattractant protein-1 and granulocyte–macrophage colony-stimulating factor (*P *< 0.05).

**Conclusion:**

Morbidity and mortality in burned patients is burn size dependent, starts at a 60% TBSA burn and is due to an increased hypermetabolic and inflammatory reaction, along with impaired cardiac function.

## Introduction

The stress response to burn injury is similar to severe trauma or critical care but differs in its severity and duration. The inflammatory response starts immediately after trauma and persists for almost 5 weeks postburn [1]. The hypermetabolic response after a major burn is characterized by a hyperdynamic response with increased body temperature, increased oxygen and glucose consumption, increased CO_2 _production, increased glycogenolysis, increased proteolysis, increased lipolysis, and increased futile substrate cycling [2]. This response begins on the fifth day postinjury and continues up to 24 months postburn, causing the loss of lean body mass, the loss of bone density, muscle weakness, and poor wound healing [2-4]. The increased metabolic requirements cause tissue catabolism, leading to nitrogen loss and a potentially lethal depletion of essential protein stores [5]. The energy requirements are met by the mobilization of proteins and amino acids. Increased protein turnover, degradation and negative nitrogen balance are all characteristic of this severe critical illness [2,5]. As a consequence, the structure and function of essential organs, such as the heart, the liver, skeletal muscle, the skin, the immune system and cellular membrane transport functions, are compromised [6-8].

Numerous studies show that an increased burn size leads to increased mortality of burned patients [9,10]. Furthermore, it was speculated that the burn size determines the extent of the hypermetabolic response. Whether mortality is due to inflammation, hypermetabolism or other pathophysiologic contributing factors is not entirely determined. The purpose of the present study was to determine in a large prospective clinical trial whether different burn sizes are associated with differences in inflammation, in body composition, in protein synthesis, and in organ function.

## Patients and methods

### Participants

All thermally injured children over a time period of 9 years who were admitted to our burns unit and required at least one surgical intervention were included in the study. Patients were resuscitated according to the Galveston formula using Ringer's lactate. Within 48 hours of admission all patients underwent total burn wound excision, in which the wounds were covered with the available autograft skin and an allograft was used to cover any remaining open areas. After the first operative procedure it was 5–10 days until the donor site was healed, and patients were then taken back to the operating theater. The skin graft procedure was repeated until all open wound areas were covered with autologous skin material.

All patients underwent the same nutritional treatment to a standardized protocol. We used the Galveston formulas – Galveston Infant, Galveston Revised, and Galveston Adolescent. The formula changes with age based on the body surface alterations that occur with growth. The intake is approximately calculated as 1,500 kcal/m^2 ^body surface area + 1,500 kcal/m^2 ^area burn. The composition of the nutritional supplement is also important. The optimal dietary composition contains 1–2 g/kg/day protein, which provides a calorie-to-nitrogen ratio of approximately 100:1 with the suggested caloric intakes. Nonprotein calories can be given either as carbohydrate or as fat, with clinical advantages for the carbohydrates. The diet was delivered by enteral nutrition, if possible, in all our patients. Total parenteral nutrition was only used as a supplemental form of nutrition when the calculated intake could not be achieved.

Patient demographics (age, date of burn and admission, sex, burn size and depth of burn) and concomitant injuries, such as inhalation injury, sepsis, morbidity and mortality, were recorded. Sepsis was defined as a blood culture identifying the pathogen during hospitalization or at autopsy, in combination with leucocytosis or leucopenia, hyperthermia or hypothermia, and tachycardia. Wound healing was evaluated from the time of donor site healing, and therefore from the time between operative interventions.

### Indirect calorimetry

As part of our routine clinical practice, all patients underwent resting energy expenditure (REE) measurements within 1 week following hospital admission, at 2–4 weeks after hospital admission, at discharge, and at 6 months postburn. Measurements of REE were performed between midnight and 5:00 am while the patients were asleep and receiving continuous feeding. The REE was measured using a Sensor-Medics Vmax 29 metabolic cart (Sensor-Medics, Yorba Linda, CA, USA). Subjects were tested in a supine position while under a large, clear, ventilated hood. The REE was calculated from the oxygen consumption and carbon dioxide production using equations described by Weir [11] All REE measurements were made at ambient temperatures of 30°C, which is the standard environmental setting for all patient rooms in our acute burn intensive care unit.

The REE measurements were used to guide nutritional management and to assess the level of metabolism. The discharge REE measurement was used to determine the level of hypermetabolism when the burn wounds were 95% healed and was included as part of the study. Measured values were compared with predicted norms based upon the Harris–Benedict equation [12]. The REE studies were repeated at 6, 9 and 12 months postburn when the patients returned for outpatient surgery. Assessments of the REE at these time points were completed utilizing the methodology and environmental settings as described above. For statistical comparison, energy expenditure was expressed both as the absolute REE and as the percentage of the basal metabolic rate predicted by the Harris–Benedict equation.

### Muscle protein net balance

The muscle protein net balance was calculated from the product of the amino acid concentration difference and the blood flow, as previously published [13-16].

### Body composition

The height and the body weight were determined clinically 5 days after admission and at discharge. The total body lean mass, fat, bone mineral density, and bone mineral content were measured by dual-energy X-ray absorptiometry. The Hologic model QDR-4500W DEXA (Hologic Inc., Waltham, MA, USA) was used to measure the body composition. To minimize systematic deviations, the Hologic system was calibrated daily against a spinal phantom in the anteroposterior, lateral, and single-beam modes. Individual pixels were calibrated against a tissue bar phantom to determine whether the pixel was reading bone, fat, lean tissue, or air. Plain anterior–posterior and lateral tibia–fibula X-rays were taken of each subject at each follow-up period to evaluate for possible premature closure of epiphyseal plates induced by anabolic agents.

### Serum hormones, proteins, and cytokines

Blood or urine was collected from the burn patients at the time of admission, preoperatively, and 5 days postoperatively for 4 weeks for serum hormone, protein, cytokine and urine hormone analysis. Blood was drawn in a serum-separator collection tube (BD, Franklin Lakes, NJ, USA) and was centrifuged for 10 minutes at 1,300 × *g*. The serum was then removed and stored at -70°C until assayed.

Serum hormones and acute phase proteins were determined using high-performance liquid chromatography and ELISA techniques. The Bio-Plex Human Cytokine 17-Plex panel was used with the Bio-Plex Suspension Array System (Bio-Rad, Hercules, CA, USA) to profile expression of 17 inflammatory mediators: IL-1β, IL-2, IL-4, IL-5, IL-6, IL-7, IL-8, IL-10, IL-12p70, IL-13, IL-17, granulocyte colony-stimulating factor, granulocyte–macrophage colony-stimulating factor, IFNγ, monocyte chemoattractant protein-1, macrophage inflammatory protein-1β, and TNF. The assay was performed according to the manufacturer's instructions. Briefly, serum samples were thawed and then centrifuged at 1,300 × *g *for 3 minutes at 4°C. Serum samples were then incubated with microbeads labeled with specific antibodies to one of the aforementioned cytokines for 30 minutes. Following a washing step, the beads were incubated with the detection antibody cocktail (each antibody specific to a single cytokine). After another washing step, the beads were incubated with streptavidin–phycoerythrin for 10 minutes, were washed again, and the concentrations of each cytokine were then determined using the array reader.

Urine cortisol was determined by standard laboratory techniques, measuring for the urine amount, creatinine and creatinine clearance.

### Liver and cardiac changes

Ultrasound measurements in this study were made with the HP Sonos 100 CF echocardiogram (Hewlett Packard Imaging Systems, Andover, MA, USA). To obtain the ultrasound liver weight, a 3.5 MHz transducer was placed directly below the midline of the rib cage on the right upper quadrant on a vertical line running through the right nipple with the patient in the supine position. Once the liver was visualized, measurements were made by scanning in a plane perpendicular to the base of the liver. The base of the liver, as well as the free edge hepatic dome, was marked on the display screen and the distance between these two points was automatically measured.

The formula used for estimating the liver weight from the single longitudinal scan along the right nipple line was weight = (1.15 *l*)^3^*d*, where *l*^3 ^represents the volume of a cube cut in half diagonally to visualize the approximate shape of the normal liver *in situ*. A factor of 1.15 was used to correct for the portion of the liver (15%) lateral to the left nipple line and representing the most inferior point of the liver. This correction was estimated from the liver at autopsy. The density (*d*) of the liver was measured on several sections by water displacement. Determining the right nipple line was not problematic unless the nipple was obliterated by a severe burn to the thorax. In these cases an approximation was made and recorded as such. The actual size was then compared with the predicted size.

M-mode echocardiograms were completed as follows. At the time of the study, none of the patients presented with or previously suffered from other concomitant diseases affecting cardiac function, such as diabetes mellitus, coronary artery disease, longstanding hypertension, or hyperthyroidism. The study variables included the resting cardiac output, the cardiac index, the stroke volume, the resting heart rate and the left ventricular ejection fraction. The stroke volume and cardiac output were adjusted for body surface area and were expressed as indexes. All ultrasound measurements were made with the Sonosite Titan echocardiogram, with a 3.5 MHz transducer. Recordings were performed with the subjects in a supine position and breathing freely. M-mode tracings were obtained at the level of the tips of the mitral leaflets in the parasternal long axis position and measurements were performed according to the American Society of Echocardiography recommendations. Left ventricular volumes determined at end diastole and end systole were used to calculate the ejection fraction, the stroke volume, the resting cardiac output and the cardiac index. Three measurements were performed and averaged for data analysis.

### Ethics and statistics

The study was reviewed and approved by the Institutional Review Board of the University Texas Medical Branch, Galveston, TX, USA. Prior to the study, each subject, parent or child's legal guardian signed a written informed consent form. Analysis of variance with post-hoc Bonferroni correction, paired and unpaired Student's *t *tests, chi-square analysis, and Mann–Whitney tests were used. Data are expressed as the mean ± standard deviation in the tables or as the mean ± standard error of the mean in the figures. Significance was accepted at *P *< 0.05.

## Results

### Demographics

One hundred and eighty-nine severely burned children were included in the present study. The patients' demographics are presented in Table 1. There was no significant difference in age and in the gender distribution between the different burn sizes (Table 1).

**Table 1:** Patient demographics

	<40% TBSA burn group (*n *= 43)	40–59% TBSA burn group (*n *= 79)	60–79% TBSA burn group (*n *= 46)	>80% TBSA burn group (*n *= 21)
Age (years)	7.3 ± 0.8	7.9 ± 0.8	7.4 ± 0.7	8.8 ± 1.3
Gender (female/male)	19/24	31/48	19/27	7/14
Time to admission (days)	9 ± 2	7 ± 1	6 ± 1^†^	3 ± 1*
Length of stay (days)	20 ± 2	26 ± 2	45 ± 5^†^	70 ± 12*
TBSA (%)	34 ± 1	50 ± 1**	70 ± 1^†^	87 ± 1*
Third-degree burn (%)	24 ± 2	36 ± 2**	60 ± 2^†^	75 ± 5*
Length of stay/% TBSA (days/%)	0.55 ± 0.05	0.53 ± 0.03	0.64 ± 0.05^†^	0.79 ± 0.13*
Operations (*n*)	2 ± 0.2	3 ± 0.2	6 ± 0.5^†^	8 ± 1*
Inhalation injury (%)	30	28	46	71*
Ventilator (%)	0	10	15^†^	35*
Infection (%)	5	18	15	19*
Sepsis (%)	0	5	24^†^	38*
Mortality (%)	0	0	13^†^	29*

Data presented as the mean ± standard deviation or percentage. TBSA, total body surface area. * Significant difference between the >80% TBSA burn group compared with the <40% and 40–59% TBSA burn groups (*P *< 0.05). **Significant difference between 40–59% TBSA versus <40% TBSA burn group, *P *< 0.05. ^†^Significant difference between the 60–79% TBSA burn group compared with the <40% and 40–59% TBSA burn groups (*P *< 0.05).

The average time from burn to hospital admission was significantly shorter in the >80% TBSA burn group and in the 60–79% TBSA burn group when compared with the other two groups (*P *< 0.05) (Table 1). The length of hospital stay was shortest in the <40% TBSA burn group, followed by the 40–59% TBSA burn group, and was longest in the large burn size groups (60–79% and >80% TBSA burn groups, respectively) (*P *< 0.05) (Table 1). Similarly, the length of stay divided by the burn size was longest in the >80% TBSA burn group, followed by the 60–79% TBSA burn group (*P *< 0.05). There was no difference between the <40% TBSA burn group and the 40–59% TBSA burn group. The number of operations was greatest in the larger burn groups followed by the 60–79% TBSA, 40–59% TBSA and <40% TBSA required the least amount of surgeries. The >80% TBSA burn group had significantly more patients with an inhalation injury and ventilator requirement compared with the other three groups (*P *< 0.05). Similarly, infection, sepsis and mortality was highest in the large burns group, followed by the 60–79% TBSA burn group and then the other two smaller burn groups (*P *< 0.05).

### Indirect calorimetry

**Figure 1:**
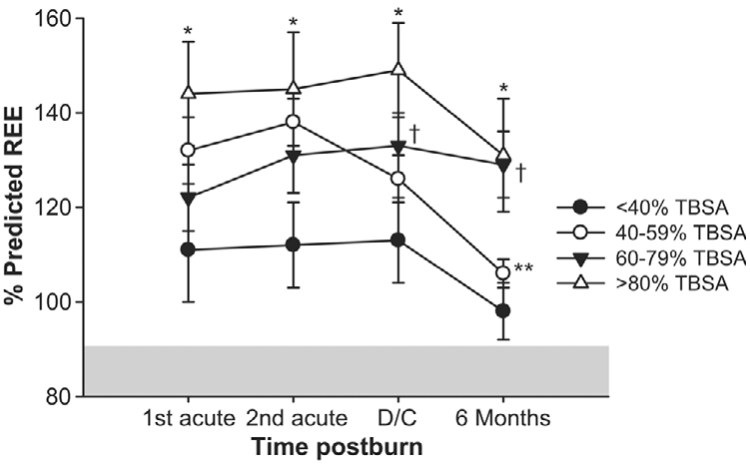
Percentage predicted resting energy expenditure. The highest percentage predicted resting energy expenditure (REE) was in children with burn >80% of their total body surface area (TBSA), followed by 60–79% TBSA burns. Children with large burns demonstrated persistent elevated percentage predicted REEs at 6 months postburn, while children with smaller burns approached the normal range. Measurements were performed at week 1 (1st acute), weeks 2–4 (2nd acute), discharge (D/C), and at 6 months postburn. *Significant difference between >80% TBSA burn group versus <40% TBSA burn group, *P *< 0.05. **Significant difference between >80% and 60–79% TBSA burn groups versus 40–59% TBSA burn group, *P *< 0.05. ^†^Significant difference between 60–79% TBSA burn group versus <40% TBSA burn group, *P *< 0.05.

The percentage predicted REE was significantly different between the four groups. Children suffering from burns of <40% TBSA had only a slight increase in the percentage predicted REE and returned to the normal range at 6 months postburn (Figure 1). Children with 40–59% TBSA burn and 60–79% TBSA burn had a significantly increased percentage predicted REE compared with children with <40% TBSA burns (*P *< 0.05) (Figure 1). The highest percentage predicted REE was in children with burns of >80% TBSA (*P *< 0.05) (Figure 1). Children with large burns demonstrated persistent elevated percentage predicted REEs at 6 months postburn (Figure 1). This indicates persistent hypermetabolism in children with burns of >60% TBSA at least up to 6 months postburn.

### Peripheral muscle protein net balance

**Figure 2:**
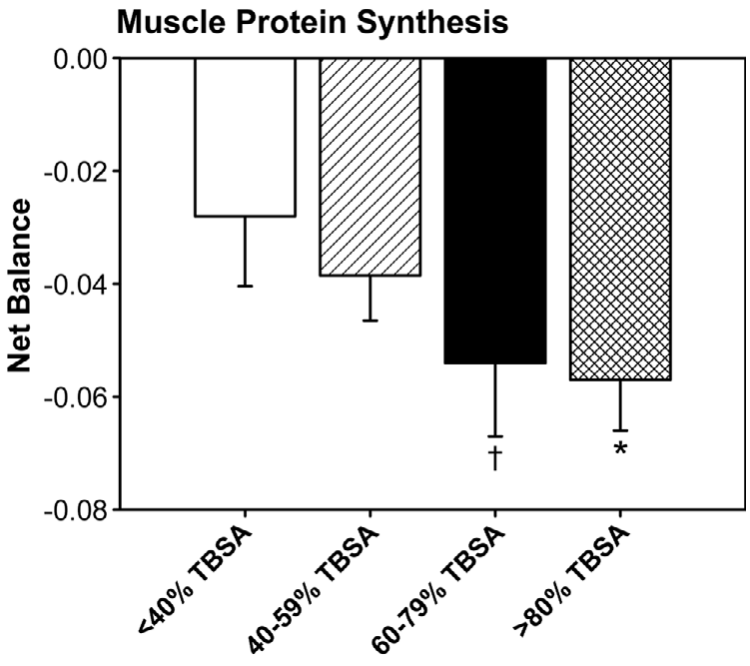
Peripheral muscle protein net balance during acute hospitalization. All burned children had a negative net protein balance in skeletal muscle. The greatest muscle protein loss was found in children with 60–79% and >80% total body surface area (TBSA) burn. *Significant difference between >80% TBSA burn group versus <40% TBSA burn group, *P *< 0.05.

All burned children had a negative net protein balance in skeletal muscle. The greatest muscle protein loss was found in children with 60–79% TBSA burns and >80% TBSA burns, indicating increased hypermetabolism and catabolism in patients sustaining a burn of >60% TBSA (*P *< 0.05) (Figure 2).

### Body composition

**Figure 3:**
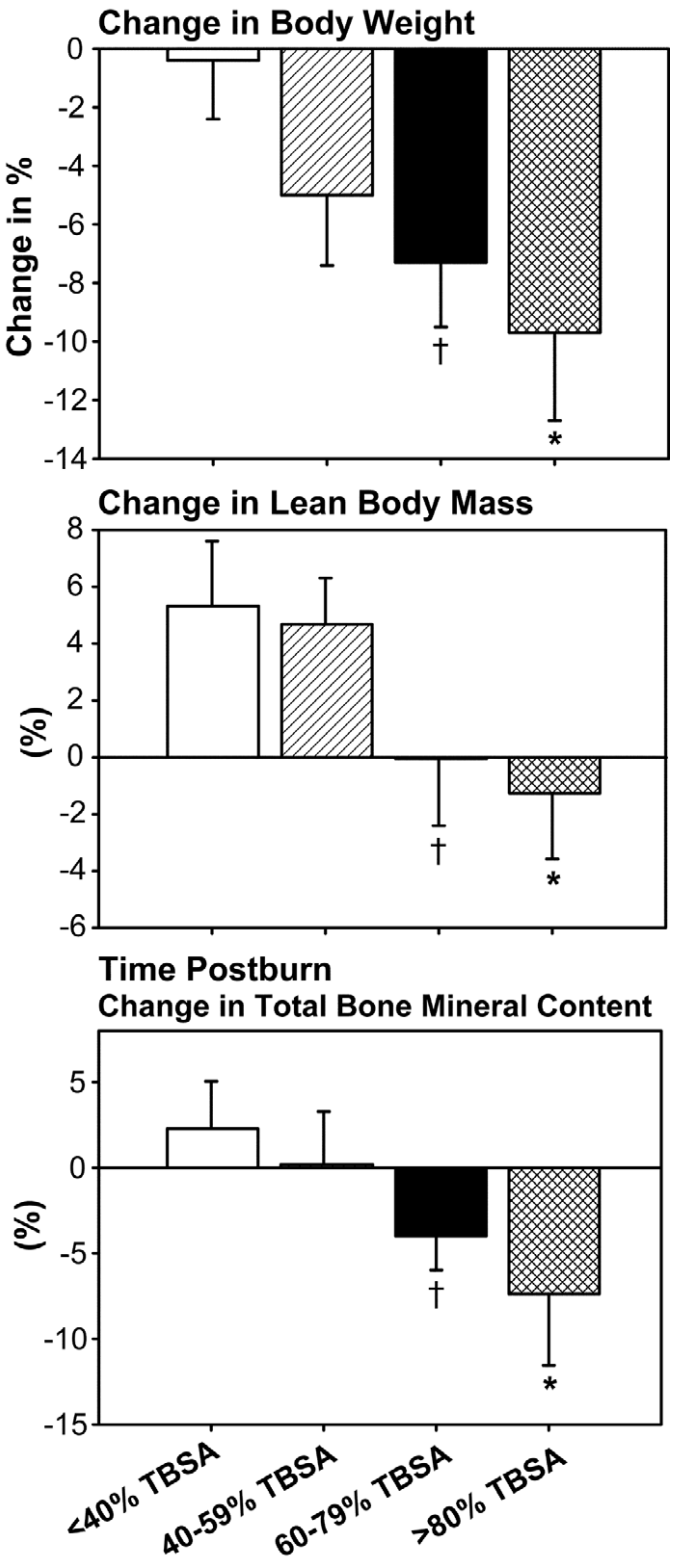
Change in body composition from admission to discharge. Children with >80% total body surface area (TBSA) burn lost the most body weight, lean body mass and bone mineral content compared with the other groups. The 60–79% TBSA burn group showed a significant loss in body weight, lean body mass and bone mineral content that was significant compared with the <40% and 40–59% TBSA groups. The <40% and 40–59% TBSA burn groups had almost no loss in body weight, lean body mass and bone mineral content. *Significant difference between >80% TBSA burn group versus <40% TBSA burn group, *P *< 0.05. ^†^Significant difference 60–79% TBSA burn group versus <40% TBSA burn group, *P *< 0.05.

There were distinct differences in body composition between the four burn groups. Children with >80% TBSA burns lost the most body weight, lean body mass and bone mineral content compared with the other groups (*P *< 0.05) (Figure 3). The 60–79% TBSA burn group showed a significant loss in body weight, lean body mass, and bone mineral content that was significant compared with the <40% and 40–59% TBSA burn groups (*P *< 0.05) (Figure 3). The <40% and 40–59% TBSA burn groups presented almost no loss of body weight, of lean body mass, and of bone mineral content (Figure 3).

### Serum hormones, proteins and cytokines

**Figure 4:**
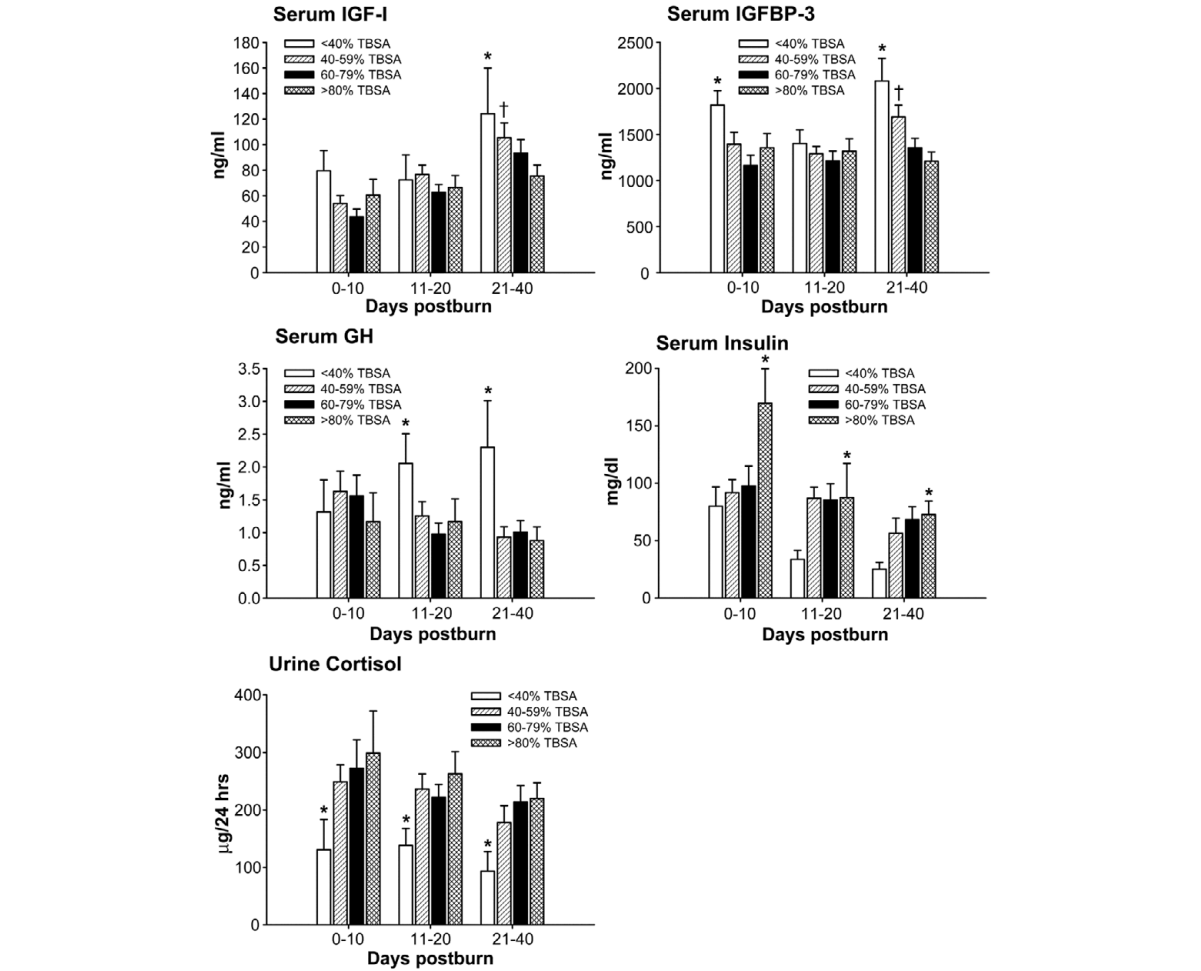
Serum protein concentrations during acute hospitalization at multiple time points. Serum insulin growth factor-I (IGF-I) was significantly increased in the <40% and 40–59% total body surface area (TBSA) burn groups compared with the 60–79% and >80% TBSA burn groups 21–40 days postburn. At the later time point, serum insulin like growth factor binding protein-3 (IGFBP-3) was significantly higher in the <40% and 40–59% TBSA burn groups when compared with the other two burn groups. Serum growth hormone (GH) markedly decreased in burns of >40% TBSA and was significantly higher in burns of <40% TBSA. Serum insulin was significantly increased in large burns immediately after burn compared with the small burns. Urine cortisol was increased during the acute stay in all four groups. Urine cortisol, however, was significantly lower in the <40% TBSA burn group when compared with the other three groups. *Significant difference between >80% TBSA burn group versus <40% TBSA burn group, *P *< 0.05. ^†^Significant difference 60–79% TBSA burn group versus <40% TBSA burn group, *P *< 0.05. Normal levels: IGF-I, 220–260 pg/ml; IGFBP-3, 3,800–4,200 pg/ml; GH, 6 pg/ml; insulin, 10–25 mg/dl; and urine cortisol, 20–45 μg/24 hours.

Serum insulin growth factor-I (IGF-I) decreased immediately after burn in all four groups (Figure 4). In the <40% TBSA and 40–59% TBSA burn groups, serum IGF-I increased over time and was significantly increased compared with the 60–79% and >80% TBSA burn groups 21–40 days postburn (*P *< 0.05) (Figure 4). Serum insulin like growth factor binding protein-3 (IGFBP-3) decreased with a burn, but its decrease was significantly attenuated in the <40% TBSA burn group (*P *< 0.05). At the later time point, serum insulin like growth factor binding protein-3 levels were significantly higher in the <40% TBSA and 40–59% TBSA burn groups when compared with the other two burn groups (*P *< 0.05) (Figure 4). Serum growth hormone markedly decreased in burns of >40% TBSA and was significantly higher in burns of <40% TBSA (*P *< 0.05) (Figure 4). Serum insulin was significantly increased in large burns immediately after burn compared with the small burns (*P *< 0.05) (Figure 4). Insulin decreased over time in all groups.

Urine cortisol was increased during the acute stay in all four groups. The urine cortisol level, however, was significantly lower in the <40% TBSA burn group when compared with the other three groups (*P *< 0.05) (Figure 4).

**Figure 5:**
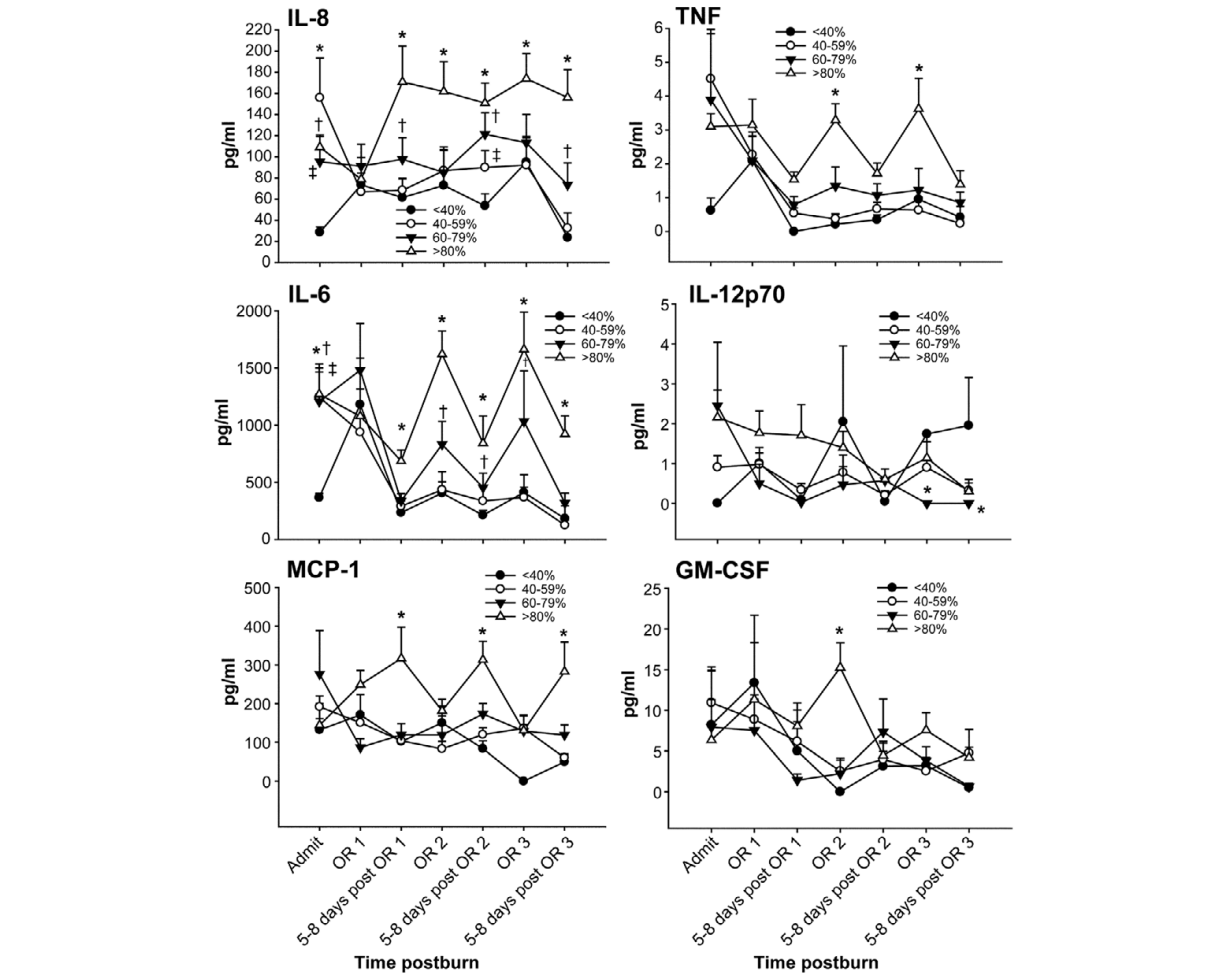
Cytokine concentrations in relation to acute hospitalization. Cytokines were measured at admission (Admit), at first surgery (OR1), at 5–8 days postsurgery 1, at second surgery (OR2), at 5–8 days postsurgery 2, at third surgery (OR3), and again at 5–8 days postsurgery 3. Six cytokines out of 17 measured were significantly different between different burn sizes. Serum IL-8, TNF, IL-6, IL-12p70, monocyte chemoattractant protein-1 (MCP-1) and granulocyte–macrophage colony-stimulating factor (GM-CSF) were significantly increased in the large burns compared with the other three groups. *Significant difference between >80% TBSA burn group versus 60–79%, 40–59% and <40% TBSA burn groups, *P *< 0.05. ^†^Significant difference between 60–79% TBSA burn group versus 40–79% and <40% TBSA burn groups, *P *< 0.05. ^‡^Significant difference between 40–59% TBSA burn group versus <40% TBSA burn group, *P *< 0.05. Normal levels: IL-8, 7.6 ± 3.9 pg/ml; TNF, 0.7 ± 0.07 pg/ml; IL-6, 8.7 ± 4.9 pg/ml; IL-12p70, 0 ± 0 pg/ml; MCP-1, 42 ± 5 pg/ml; and GM-CSF, 0 ± 0 pg/ml.

In terms of serum cytokines, we found that only six cytokines were significantly and possibly clinically relevantly affected. Serum IL-8, TNF, IL-6, IL-12p70, monocyte chemoattractant protein-1 and granulocyte–macrophage colony-stimulating factor were significantly increased in the large burns. In general, there was a notion that the smaller the burn size, the lower the cytokine concentration – indicating a relationship between burn size and cytokine expression (Figure 5).

Multiple cytokines, such as IL-1β, macrophage inflammatory protein-1β, IFNγ, IL-10, granulocyte colony-stimulating factor, IL-17, IL-13, IL-7, IL-5, IL-4, and IL-2, were affected by the burn but not by the burn size. Cytokines increased with the injury and decreased over time. These cytokines showed no significant difference between the burn size groups, but presented significant changes over time.

### Liver and cardiac changes

**Figure 6:**
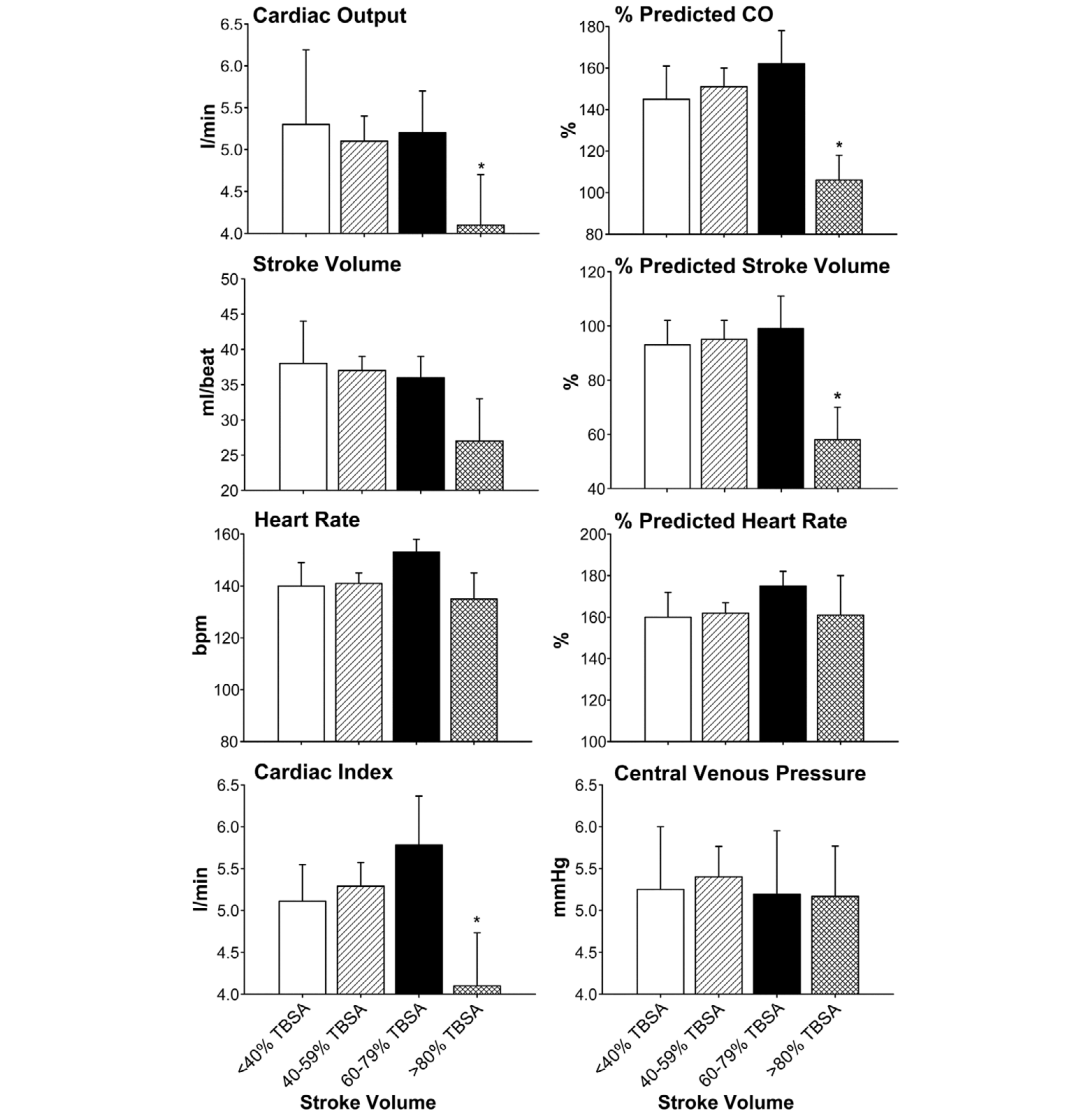
Cardiac function within 96 hours after hospital admission. The cardiac output and the predicted cardiac output (predicted CO) were significantly decreased in burns of >80% total body surface area (TBSA), while there was no difference between the three other burn groups. The predicted stroke volume was significantly decreased in the >80% TBSA burn group when compared with the other three groups. There were, however, no differences between groups in the heart rate, predicted heart rate, cardiac index, and central venous pressure. *Significant difference between >80% TBSA burn group versus 60–79%, 40–59% and <40% TBSA burn groups, *P *< 0.05.

Analysis of the cardiac output, the predicted cardiac output, the stroke volume, the predicted stroke volume, the heart rate, the predicted heart rate, the cardiac index, and the central venous pressure showed differences between groups. The cardiac output and the predicted cardiac output were significantly decreased in the burns >80% TBSA (*P *< 0.05), while there was no difference between the three other burn groups (Figure 6). The predicted stroke volume was also significantly decreased in the >80% TBSA burn group when compared with the other three groups (*P *< 0.05) (Figure 6). There were, however, no differences between groups in the heart rate, the predicted heart rate, the cardiac index, and the central venous pressure (Figure 6).

**Figure 7:**
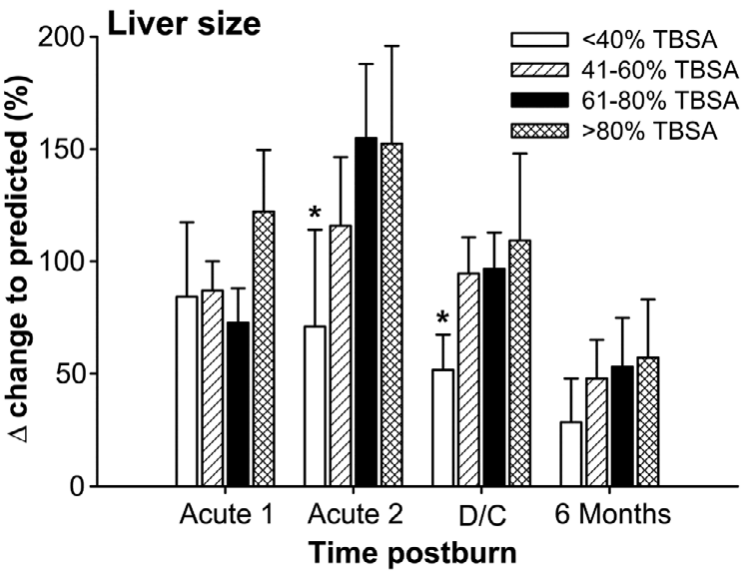
Liver size increased by almost 100% immediately after burn in all groups. The liver size was significantly smaller in <40% total body surface area (TBSA) burns compared with the other groups. *Significant difference between <40% TBSA burn group versus 40–59%, 60–79% and >80% TBSA burn groups, *P *< 0.05. Measurements were performed at week 1 (Acute 1), at weeks 2–4 (Acute 2), at discharge (D/C), and at 6 months postburn.

Immediately after burn, the liver size increased by almost 100% in all groups (Figure 7). While the liver in burns of >60% TBSA further increased in size to 150%, the liver size in burns of <40% TBSA decreased rapidly (*P *< 0.05) (Figure 7). There was no significant difference in liver size increase between burns of 40–59% TBSA, of 60–79% TBSA, and of >80% TBSA (Figure 7).

## Discussion

The metabolic rate in burns is extremely high, and energy requirements are immense and are met by the mobilization of proteins and amino acids [5]. As a consequence, the structure and function of essential organs such as skeletal muscle, skin, immune system, and cellular membrane transport functions are compromised [17-19]. Catecholamines involved in the hypermetabolic response to burn injury are released from sympathetic nerve ends and the adrenal medulla, and are raised two-fold to 10-fold in proportion to the burn size [20-23]. In the present study, by comparing four different burn sizes, we showed that an increase in burn size is associated with increased hypermetabolism, with persistent inflammation, with catabolism, with changes in body composition, with increased stress hormone production, and with organ dysfunction. Whether the correlation between these phenomena and burn size is linear was not determined, but we clearly observed these pathophysiologic changes with increased burn size.

Large burns cause a marked increase in inflammation and catecholamines, which leads to an increased metabolic rate. In the present study, we found that smaller burn injuries demonstrated decreased catecholamine and stress hormone levels that were associated with decreased hypermetabolism and catabolism. Smaller burns had significantly decreased and shortened predicted REE, body weight loss, and net muscle protein balance when compared with the larger burns. Further indicators that smaller burns are less hypermetabolic than larger burns are decreased acute stress hormones and increased anabolic hormones. Catecholamines, cytokines and proinflammatory mediators are known to block, and therefore decrease, endogenous anabolic agents via cellular mediators [24,25].

We showed in the present study that patients with large burns demonstrate a different cytokine expression profile compared with patients with smaller burn injuries. Patients with burns over 80% TBSA had persistent and marked increased levels of IL-8, IL-6, monocyte chemoattractant protein-1 and TNF. All of these cytokines are proinflammatory and enhance catabolism and hypermetabolism via hyperinflammation. We therefore suggest that these high levels of proinflammatory cell mediator trigger and enhance the hypermetabolic response with increased stress hormones and protein catabolism. On the other hand, lower inflammation and hypermetabolism was associated with higher endogenous anabolic hormone levels. Patients with smaller burns had lower inflammatory marker and stress hormones, which was associated with higher IGF-I, insulin like growth factor binding protein-3 and growth hormone levels. IGF-I was shown to exert anabolic effects on skeletal muscle protein synthesis, to attenuate the hepatic acute phase response, and to improve dermal and epidermal regeneration [26-30]. Furthermore, decreased growth hormone and IGF-I levels lead to a deficit in transmembrane amino acid transport and a compromised immune system [31-35].

The glucose kinetics in severely burned patients is almost always abnormal [36,37]. Glucose utilization in burned patients is almost entirely through inefficient anaerobic mechanisms, as characterized by increased lactate production, which accounts for increased glucose consumption [22,23,38]. Glucose production, particularly from alanine, is elevated in almost all patients with severe burn [5]. The increased gluconeogenesis from amino acids renders these amino acids unavailable for reincorporation into body protein. Nitrogen is excreted, primarily in urea, thus contributing to the progressive depletion of body protein stores. Plasma insulin levels usually remain normal or slightly elevated in burn patients [39,40]. The fact that the basal rate of glucose production is elevated despite normal or elevated plasma insulin levels indicates hepatic insulin resistance, since under normal conditions elevated serum insulin would lower the rate of glucose production [38-40]. Furthermore, the plasma glucose concentration is frequently increased, which would normally directly inhibit glucose production [41,42]. As hyperglycemia is associated with increased mortality in critically ill patients [43,44] and worsens the outcome in severely burned patients [41,42], we determined serum insulin levels in our four burn groups. We found that insulin levels were significantly increased in the >80% TBSA burn group and were lower in the smaller burn groups. This indicates that with the increased severity of the burn injury insulin resistance increases and more insulin needs to be synthesized to maintain normoglycemia.

Another striking finding of this study was the cardiac function. Burn patients with burns of >80% TBSA demonstrated significantly decreased cardiac output and percentage predicted cardiac output. Furthermore, these patients had significantly decreased percentage predicted stroke volumes and cardiac index when compared with the other groups. There were no differences in the heart rate, the predicted heart rate, and the central venous pressure between groups. As the preload was not significantly different between groups, we therefore suggest that a severe burn over 80% TBSA causes a marked myocardial depression. A myocardial depression associated with a severe burn injury has been shown in several studies [45-47]. These data demonstrate that myocardial depression plays an important role during the postburn response and that modulation of myocardial depression may improve clinical outcome. The hypothesis that myocardial dysfunction may be one of the main contributors to mortality in large burns was confirmed in a recent retrospective autopsy study [10].

## Conclusion

Based on our findings, we suggest that a burn injury involving more than 80% of the total body surface causes marked and prolonged inflammation, marked increases in hypermetabolism, catabolism and cardiac dysfunction, and, subsequently, higher incidences of infection, sepsis and death. Treatment should focus on several aspects of the pathophysiologic events postburn, such as treatment of the inflammatory response, insulin resistance, hypermetabolism, catabolism, and cardiac dysfunction.

## Key messages

• The inflammatory and metabolic response is dependent on the burn size.

• Under 60% TBSA burn, the hypermetabolic and inflammatory response is less pronounced.

• Between 60% and 80% TBSA burn, patients are severely hypermetabolic and hyperinflammatory, but they do not undergo cardiac dysfunction.

• Cardiac dysfunction only occurs in burns over 80% TBSA, and patients undergo a drastic inflammatory and hypermetabolic response.

• Patients should be referred to a major burn center with a burn size over 40–60% TBSA; antinflammatory and anabolic treatment should be initiated for burn sizes of 40–60% TBSA; however, cardiac monitoring and treatment must be initiated in burns over 80% TBSA.

• Cardiac dysfunction appears one of the major contributing factors for mortality, and the treatment of cardiac dysfunction may improve survival.

## Abbreviations

ELISA = enzyme-linked immunosorbent assay; IFN = interferon; IGF-I = insulin growth factor-I; IL = interleukin; REE = resting energy expenditure; TBSA = total body surface area; TNF = tumor necrosis factor.

## Competing interests

The authors declare that they have no competing interests.

## Authors' contributions

MGJ designed the study, gathered data, conducted the statistics and wrote the manuscript. RPM collected data, reviewed the statistical analysis and reviewed the manuscript. CCF performed experiments to obtain data, conducted the statistical analysis, and helped to write the manuscript. WBN helped to collect data and write the manuscript. GAK helped to obtain data, performed and established methods to obtain serum analyses, and reviewed the manuscript. GGG collected data, reviewed the statistical analysis and reviewed the manuscript. DNH gathered data, reviewed the analysis, and helped to write the manuscript.
